# Versatility Investigation of Grown Titanium Dioxide Nanoparticles and Their Comparative Charge Storage for Memristor Devices

**DOI:** 10.3390/mi14081616

**Published:** 2023-08-16

**Authors:** Shubhro Chakrabartty, Abdulkarem H. M. Almawgani, Sachin Kumar, Mayank Kumar, Suvojit Acharjee, Alaaddin Al-Shidaifat, Alwin Poulose, Turki Alsuwian

**Affiliations:** 1 Department of Electronics and Communication Engineering, Koneru Lakshmaiah Education Foundation University (K L College of Engineering), Vaddeswaram 522302, Andhra Pradesh, India; shubhro.chakrabartty87@gmail.com; 2 Electrical Engineering Department, College of Engineering, Najran University, Najran 66439, Saudi Arabia; alaaddinsh@hotmail.com; 3 Department of Electronics and Communication Engineering, SRM Institute of Science and Technology, Kattankulathur 603203, Tamil Nadu, India; 4 Technical Research Analyst (TRA), Electronics/Biomedical Engineering, Aranca, Mumbai 400076, Maharashtra, India; mayank.kumar@aranca.com; 5 Department of Electronic and Communication Engineering, Narula Institute of Technology, Agarpara, Kolkata 700109, West Bengal, India; acharjeesuvo@gmail.com; 6 Department of Nanoscience and Engineering, Centre for Nano Manufacturing, Inje University, Gimhae 50834, Republic of Korea; tmalsuwian@nu.edu.sa; 7 School of Data Science, Indian Institute of Science Education and Research Thiruvananthapuram (IISER TVM), Vithura, Thiruvananthapuram 695551, Kerala, India; alwinpoulosepalatty@iisertvm.ac.in

**Keywords:** memristor, nanoparticles, titanium dioxide, TLBO, XRD

## Abstract

Memristive devices have garnered significant attention in the field of electronics over the past few decades. The reason behind this immense interest lies in the ubiquitous nature of memristive dynamics within nanoscale devices, offering the potential for revolutionary applications. These applications span from energy-efficient memories to the development of physical neural networks and neuromorphic computing platforms. In this research article, the angle toppling technique (ATT) was employed to fabricate titanium dioxide (TiO_2_) nanoparticles with an estimated size of around 10 nm. The nanoparticles were deposited onto a 50 nm SiO_x_ thin film (TF), which was situated on an n-type Si substrate. Subsequently, the samples underwent annealing processes at temperatures of 550 °C and 950 °C. The structural studies of the sample were done by field emission gun-scanning electron microscope (FEG-SEM) (JEOL, JSM-7600F). The as-fabricated sample exhibited noticeable clusters of nanoparticles, which were less prominent in the samples annealed at 550 °C and 950 °C. The element composition revealed the presence of titanium (Ti), oxygen (O_2_), and silicon (Si) from the substrate within the samples. X-ray diffraction (XRD) analysis revealed that the as-fabricated sample predominantly consisted of the rutile phase. The comparative studies of charge storage and endurance measurements of as-deposited, 550 °C, and 950 °C annealed devices were carried out, where as-grown device showed promising responses towards brain computing applications. Furthermore, the teaching–learning-based optimization (TLBO) technique was used to conduct further comparisons of results.

## 1. Introduction

The advancement of miniaturization has revolutionized the field of electronics, enabling new possibilities [1]. Titanium dioxide (TiO_2_) is a versatile semiconductor with three distinct crystalline forms: anatase, rutile, and brookite [2]. Due to its favorable combination of physical and chemical properties, environmental compatibility, and cost-effectiveness, polycrystalline TiO_2_ has found numerous applications and holds great potential for solar cells [3], flexible electronics [4], detectors [5], etc. TiO_2_ has also garnered attention for its resistive switching capabilities [6]. Furthermore, memristor devices with TiO_2_ nanoparticles/Ag (silver) and TiO_2_ nanoparticles/Au (gold) electrodes have also been used as synaptic emulators for advanced neurocomputing applications [6].

The achievement of neuromorphic resistive memory in TiO_2_ thin films represents a significant milestone in the pursuit of future computing [7,8]. Various studies have emphasized memristivity as a common trait by electromigration of point defects in titanium oxide systems [9]. One of the key advantages of TiO_2_ films in memristors is their compatibility with existing semiconductor fabrication processes [10]. TiO_2_ can be deposited as thin films using various techniques such as atomic layer deposition, sputtering, or chemical vapor deposition, allowing for integration into conventional integrated circuit manufacturing [11]. Moreover, TiO_2_ films can be easily patterned and engineered at nanoscale dimensions, enabling the creation of densely packed memristor arrays with high device densities and improved performance. The memristive behavior of TiO_2_ films can be attributed to the presence of oxygen vacancies and defect sites within the material [11]. These defects serve as charge carriers and contribute to the modulation of resistance states. By applying electrical pulses or voltage biases, the oxygen vacancies can be controlled and manipulated, leading to the switching between high- and low-resistance states, which can be read as digital bits, forming the basis for non-volatile memory storage and computing operations [12]. In addition to their memristive properties, TiO_2_ films also exhibit exceptional endurance, retention, and scalability, which are crucial factors for the practical implementation of memristor-based technologies. Endurance refers to the ability of the device to withstand repeated switching cycles without degradation, while retention signifies the capability of maintaining stored information over long periods. TiO_2_ films have demonstrated impressive performance in these aspects, making them highly desirable for future memory and computing architectures [13].

**Figure 1:**
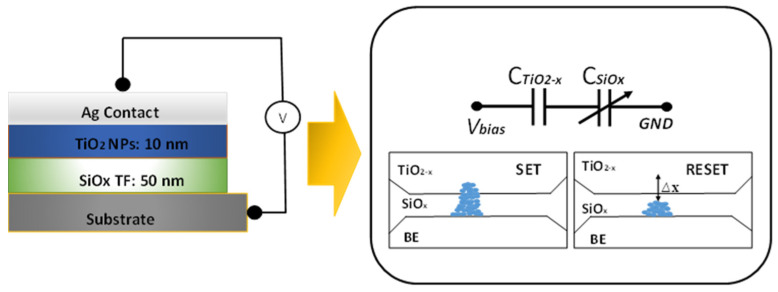
Schematic and SET and RESET process of device.

In this research article, we manufactured TiO_2_ nanoparticles through the angle topping technique. The grown nanoparticles were further annealed in two different temperatures to check their charge storage capacity. The junction capacitance of the devices was measured across a range of frequencies (from Hz to MHz) with a positive potential applied to the top Ag electrode. Additionally, the teaching–learning-based optimization (TLBO) method was successfully applied to determine the maximum charge storage capacity of the devices. The schematic representation is shown in Figure 1.

## 2. Experiment Procedure

Normal deposition was employed to deposit a 50 nm SiO_x_ thin film (TF) layer onto an n-type Si <100> substrate (1–30 ohm-cm) using an e-beam evaporator. Subsequently, the angle toppling technique (ATT) was employed to manufacture 10 nm TiO_2_ nanoparticles inside the chamber of an e-beam evaporator (Hind High Vacuum Co. (P) Ltd., 15F6, Bengaluru, India) over SiO_x_ TF. The base pressure during the deposition process was maintained at 2 × 10^−5^ mbar, and the deposition rate was monitored using a quartz crystal at a rate of 1.2 Å s^−1^. The substrate holder was positioned 24 cm away from the evaporated material source at an orientation angle of 85° with respect to the perpendicular line between the source and the substrate. The substrate also underwent an azimuthal rotation at a speed of 460 rpm.

The as-fabricated samples were subsequently annealed individually in open air conditions using a tube furnace (GSL-1700X, MTI, New York, NY, USA). The annealing process involved heating and cooling ramps of 4 °C/min and one hour of annealing time at temperatures of 550 °C and 950 °C. The surface topography of the samples was analyzed using a field emission gun-scanning electron microscope (FEG-SEM) (JEOL, JSM-7600F) with a vacuum maintained at 2.17 × 10^−9^ mBar. Chemical mapping and X-ray diffraction (XRD) analysis (using Cu Kα radiation) were also performed on the samples using equipment from Bruker (D8 Advance).

For device fabrication, a 1.5 mm diameter Ag top electrode was created on the TiO_2_ nanoparticles of the as-fabricated sample and the two annealed samples. The capacitance of the TiO_2_ nanoparticles/SiO_x_ TF-based devices was measured using an Agilent LCR meter (E4980A) with the Ag top contact.

## 3. Teaching–Learning-Based Optimization (TLBO) Methodology

The TLBO algorithm, developed in [14,15], has gained significant recognition for its effectiveness as a parameter-free optimization technique. TLBO draws inspiration from the relationship between mentors and students in a classroom scenario, harnessing population-based meta-heuristic principles to find global solutions. This algorithm simulates the teaching and learning dynamics, where a population of individuals represents the learners in TLBO.

In TLBO, the algorithm revolves around two essential components: the mentor (representing the teacher phase) and the students (representing the learner phase). The mentor guides the students, similar to a teacher imparting knowledge, while the students learn through interactions with their peers. The collective performance of the students, influenced by the mentor’s expertise, is used to evaluate the algorithm’s output. The aim is to ensure that the mentor cultivates the students’ skills, enabling them to achieve superior results in their examinations. Additionally, collaborative learning among the students contributes to their overall improvement.

One notable advantage of TLBO is its rapid convergence time, particularly when applied to problems with lower dimensions. The algorithm shares common parameters, such as population size and stopping criteria, with other heuristic optimization techniques. TLBO employs two primary phases to generate a new population: the teacher phase, where guidance is provided by the mentor, and the learning phase, where the learners interact with each other [16].

(1) $$newx_{i}=x_{i}+DM$$

(2) $$newx_{i}=x_{i}+Y\left(i\right),\;if\;f\left(x_{i}\right)<f\left(x_{j}\right)\;=x_{i}+y\left(j\right),\;otherwise$$

where *Y* is {*x_i_*^1^, *x_i_*^2^, *x_i_*^3^, …, *x_i_^m^*}, *DM* is the difference mean = *r* (Teacher − *TF* Mean), *TF* is round [1 + rand (0, 1)], *Y*(*i*) = *r*(*x_i_* − *x_j_*) and *Y*(*j*) = *r*(*X_j_* − *X_i_*).

**Figure 2:**
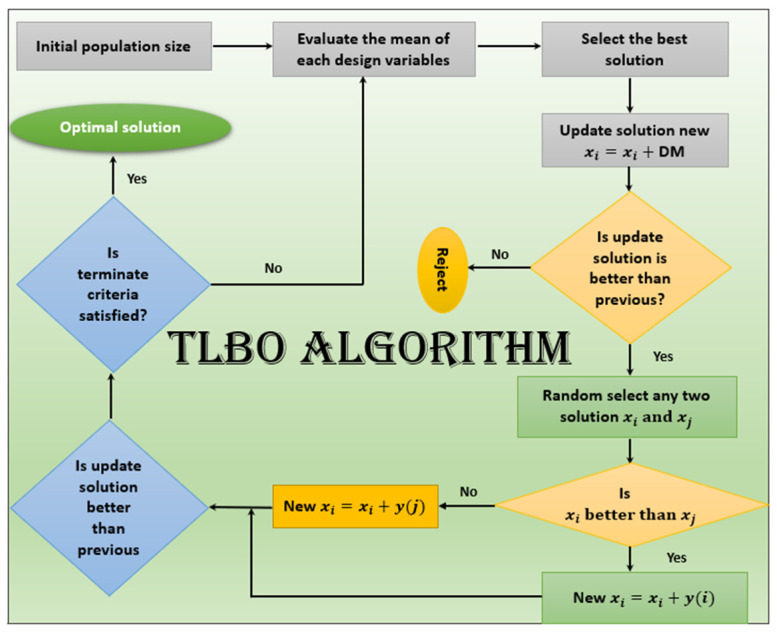
Flowchart of the proposed training scheme for TLBO algorithm.

Equation (1) depicts the advancement of students’ abilities through the guidance of mentors and the beneficial educational interactions among the students, as demonstrated in Equation (2). The process of the TLBO algorithm is illustrated in Figure 2.

Initially, we measured the junction capacitance of the devices across a wide frequency range (from Hz to MHz) by applying a positive potential to the top Ag electrode. Subsequently, an optimization technique was employed to determine the maximum charge storage in the devices under different conditions: as fabricated, 550 °C annealed, and 950 °C annealed. In this study, the objective function for optimization was defined as charge storage (*W*), which relied on the design variables including capacitance (*C*), voltage (*V*), and frequency range (Hz to MHz). Table 1 outlines the upper and lower bounds of each parameter at various frequencies.

(3) $$W=\frac{1}{2}CV^{2}$$

$$Subject\;to\;V_{min}\;\leq \;V\;\leq \;V_{max}$$

$$C_{min}\;\leq \;C\;\leq \;C_{max}$$

## 4. Results and Discussion

### 4.1. Structural Topography

**Figure 3:**
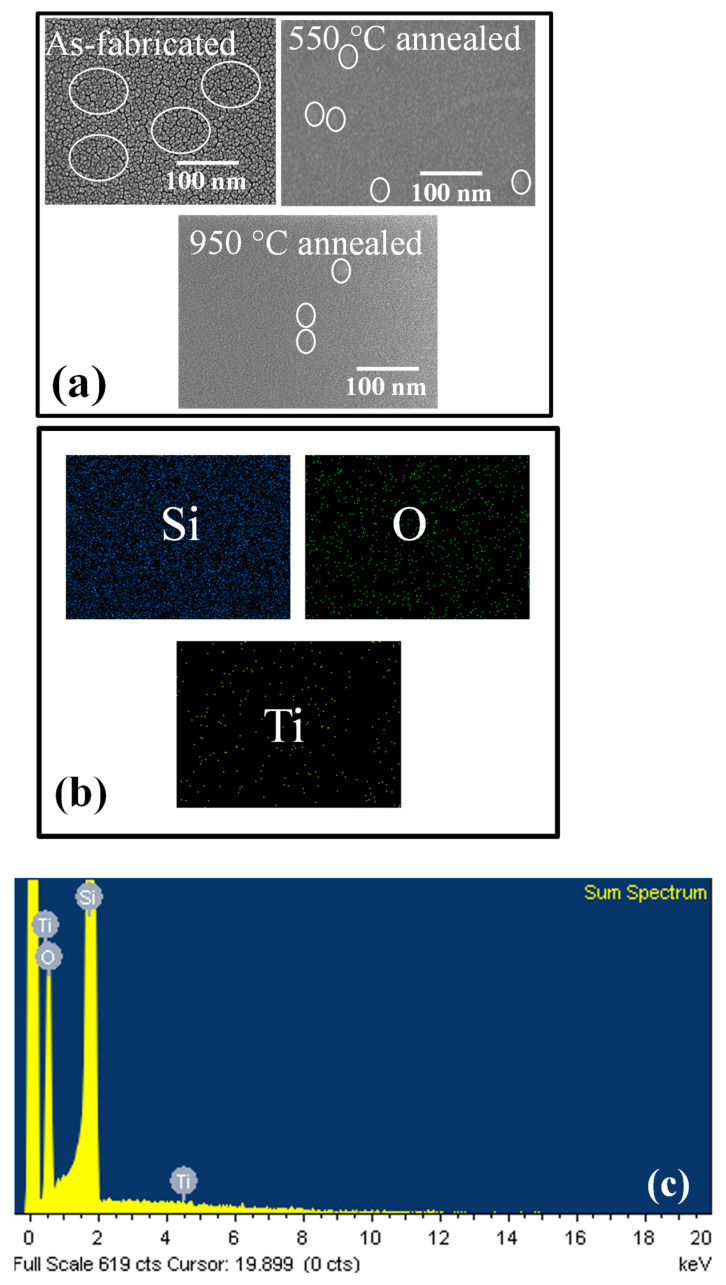
FEG-SEM images of TiO_2_ nanoparticles/SiO_x_ TF/n-type Si (as-fabricated and 550 °C and 950 °C annealed samples): (**a**) Top view, (**b**) Chemical mapping, (**c**) EDX analysis.

Figure 3a depicts the FEG-SEM image of the samples in their as-fabricated and annealed states at temperatures of 550 °C and 950 °C. Upon closer examination, it is evident that the as-fabricated sample exhibits a significantly higher presence of nano clusters (NCs) islands compared to both the 550 °C and 950 °C annealed samples. Consequently, a larger amount of charge is expected to be trapped within the charge trapping sites of nanoclusters in the as-fabricated sample in comparison to the annealed samples. The defects primarily utilized for charge trapping include dislocations and grain boundaries [17]. As the annealing temperature increases, these defects are reduced, leading to a decrease in nucleation space in the 550 °C and 950 °C annealed samples. Furthermore, the EDX analysis (Figure 3b) confirms the presence of titanium (Ti), oxygen (O_2_), and silicon (Si) in the sample. The spectrum illustrates the emission from Ti Kα1, O_2_ K α1, and Si K α1 shells, as displayed in Figure 3c.

**Figure 4:**
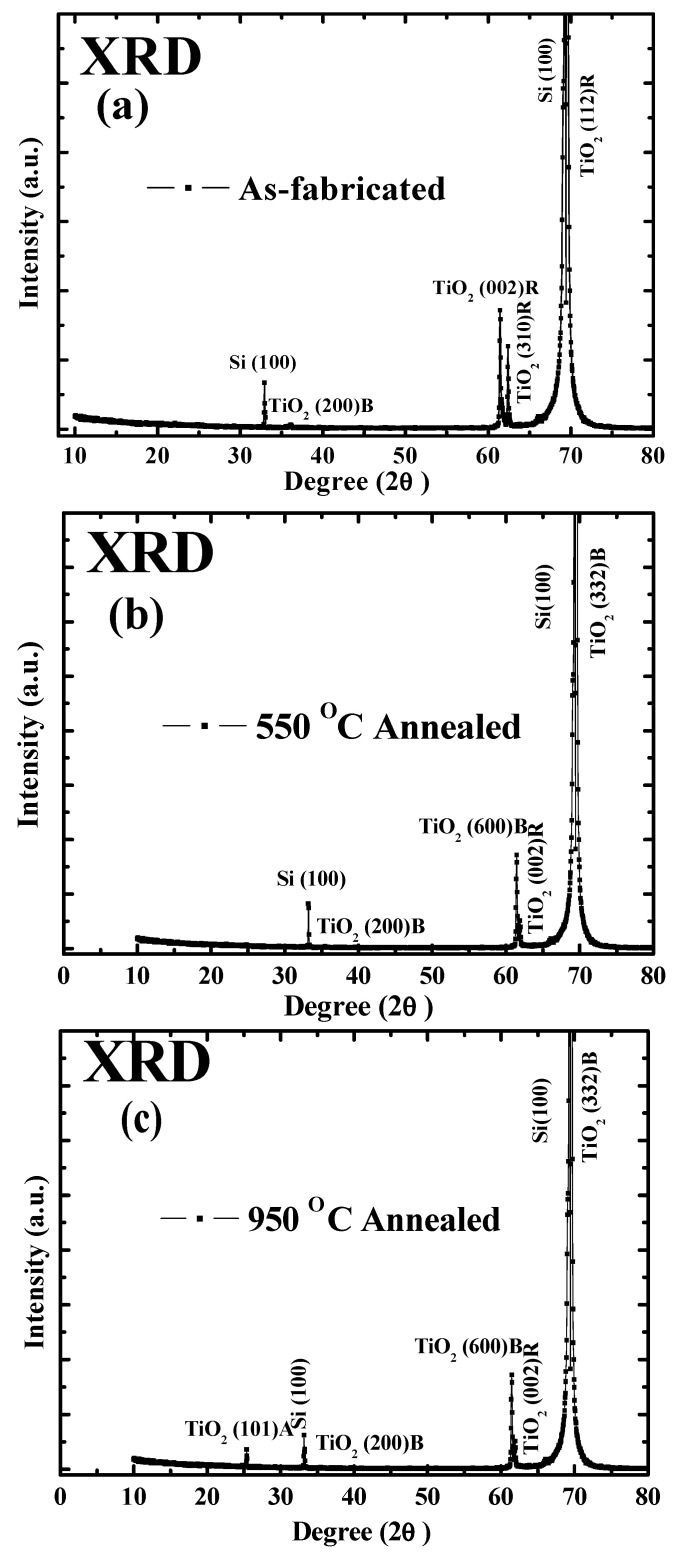
XRD pattern of as-fabricated and annealed samples: (**a**) As-fabricated, (**b**) 550 °C, (**c**) 950 °C.

Figure 4 illustrates the polycrystalline characteristics of TiO_2_ nanoparticles as revealed by X-ray diffraction. It is observed that the crystallinity of the fabricated sample increases with an increase in temperature [18]. However, the initial as-fabricated sample primarily consists of the rutile phase, which is known to be the most stable phase [19] when compared to anatase and brookite by taking reference of [20].

### 4.2. Endurance of Devices and Their Performance

**Figure 5:**
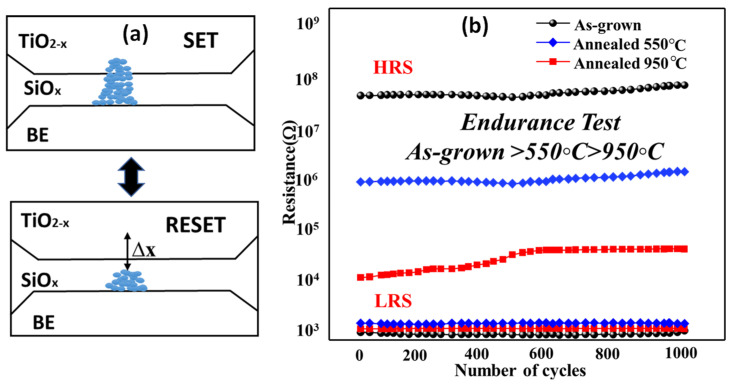
(**a**) SET and RESET process of the device, (**b**) Endurance test of the devices.

The capacitance–voltage (C–V) characteristics of the bilayer are presented in Figure 5. It depicts a cross-section illustration of Ag/TiO_2−x_/SiO_x_/Si structure and schematic circuit diagram. When a positive *V_bias_* is applied to the device, it induces the formation of a conducting filament comprising oxygen vacancies within the TiO_2_ layer (the switching layer). This filament provides a path for electron movement facilitated by the voltage distribution across the capacitance of the two dielectrics. During the RESET process, a thin dielectric barrier, denoted as Δ*x*, initiates formation at the TiO_2−x_/SiO_x_ interface, which corresponds to the thinner section of the conducting filament. This barrier effectively interrupts the conductive path as the oxygen vacancies drift back towards the bottom due to the voltage potential difference between the electrodes.

The measured retention of the low-resistance state (LRS) and high-resistance state (HRS) at room temperature shows the as-deposited devices exhibited long-lasting stability over several decades of endurance, likely referring to their ability to retain their resistive switching properties over a long period of time. The larger oxygen vacancies within the as-deposited devices play a crucial role in the resistive switching mechanism. Here, larger oxygen vacancies led to the formation of a strong conductive path within the device. This conductive path is responsible for the resistive switching behavior observed in as-deposited TiO_2_ nanoparticles/SiO_x_ device. It shows promising retention, indicating their ability to retain their resistive switching properties over time. This suggests that the devices have the potential for long-term reliability.

### 4.3. TLBO

**Figure 6:**
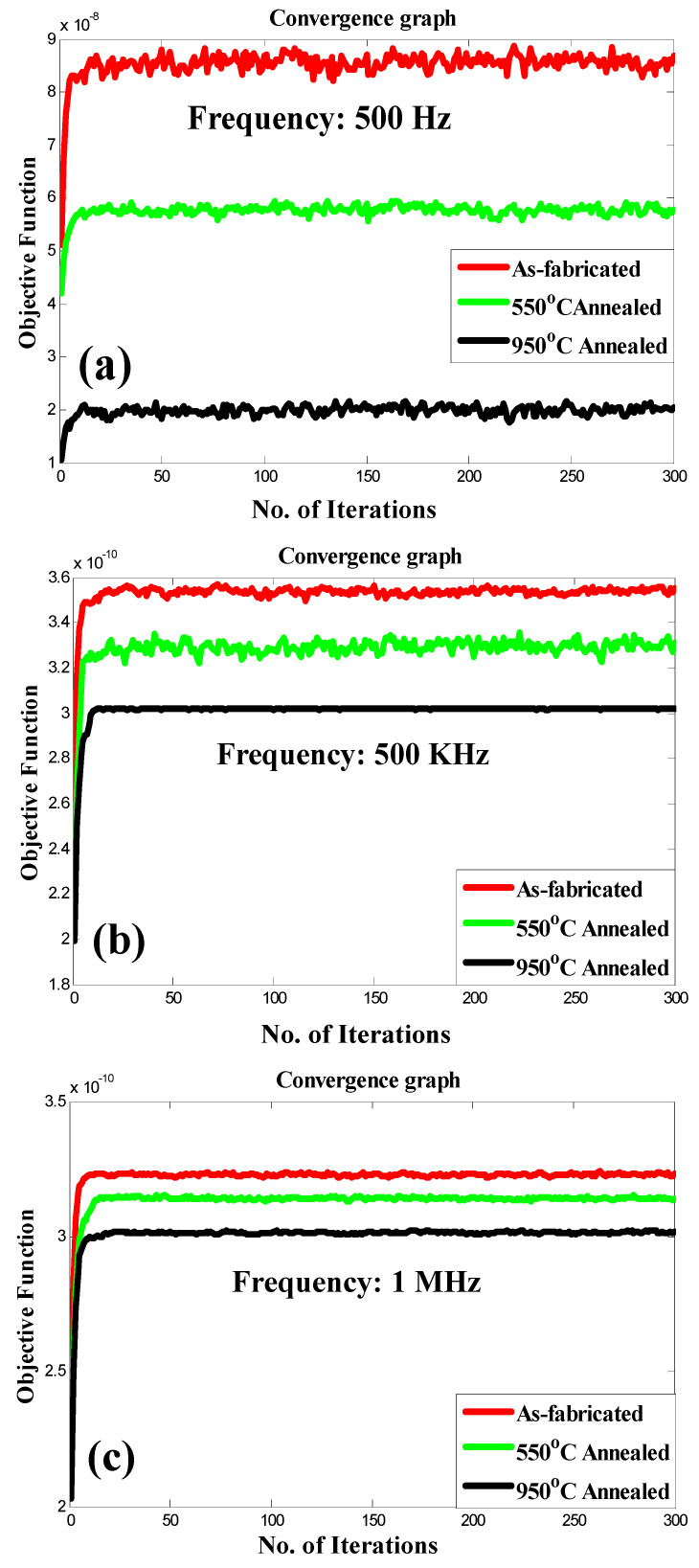

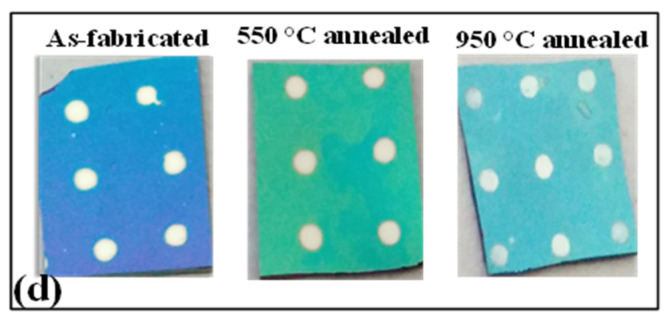
Charge storage graphs: (**a**) 500 Hz frequency, (**b**) 500 kHz frequency, (**c**) 1 MHz frequency, (**d**) device image with top Ag contact.

The TLBO technique was employed to analyze the response of the device. The measurements were conducted on devices in their as-fabricated state, as well as on those annealed at temperatures of 550 °C and 950 °C, in order to determine the charge storage. To optimize our objective function, the TLBO technique was further applied. The objective function in this study depends on two parameters, capacitance (*C*) and voltage (*V*), making it a two-dimensional problem. The convergence graphs in Figure 6a–c depict the charge storage for the as-fabricated and annealed devices. The TLBO algorithm was allowed to run for maximum 300 iterations. However, the algorithm reached convergence within 25 iterations. After reaching convergence, it can be observed that the charge storage in the as-fabricated device surpasses that of the annealed devices, thus confirming the experimental findings. After 300 iterations, it can be observed that the charge storage in the as-fabricated device surpasses that of the annealed devices, thus confirming the experimental findings. Table 2 presents the optimal values of the objective function after 300 iterations and with a population size of 50. From Figure 6a–c and Table 2, it is evident that the charge storage capability of the as-fabricated device exceeds that of the annealed devices.

From Table 2, it can be estimated that the proposed annealed devices consume energy in the pico-Joule range at optimal voltage and capacitance, whereas the as fabricated device will consume energy in the nano-Joule range at optimum voltage and capacitance. Most of the CMOS-based memory circuits consume the power in nano-Joule range [21]. Therefore, the proposed TiO_2_-based annealed device are energy efficient and can be used to construct energy efficient memory.

## 5. Conclusions

To summarize, the ATT was utilized to create TiO_2_ nanoparticles on a SiOx TF over an n-type Si substrate. Subsequently, the fabricated samples underwent annealing at 550 °C and 950 °C in ambient air. Upon examination using FEG-SEM, it was observed that the as-fabricated sample exhibited more prominent nanoclusters compared to the samples annealed at 550 °C and 950 °C. This suggests that the as-fabricated sample has a higher capacity for charge trapping. XRD analysis revealed that as the temperature increased, the samples became more crystalline. However, the as-fabricated sample predominantly displayed the rutile phase, known for its stability among other phases. Experimental measurements and the TLBO technique were employed to calculate the charge storage capacity of the as-fabricated device, as well as the devices annealed at 550 °C and 950 °C. In both cases, the as-fabricated device exhibited higher charge storage capacity compared to the annealed devices. Consequently, the TiO_2_ nanoparticles-based device fabricated through the as-fabrication process holds potential for charge trapping and the development of storage devices and exhibit stable endurance, reliable resistive switching behavior, and promising retention, making them a potential candidate for memristor devices.

## Figures and Tables

**Table 1:** Tabular representation of boundary of design variables.

	Frequency	*v_min_* (V)	*v_max_* (V)	*C_min_* (pF)	*C_max_* (pF)
As Fabricated	1 MHz	−10	10	6.3	6.52067
	500 kHz	−10	10	6.6614	7.2518
	500 Hz	−10	10	1230	1880
550 °C Annealed	1 MHz	−10	10	6.145	6.33665
	500 kHz	−10	10	5.6721	6.899
	500 Hz	−10	10	890.26	1260.13
950 °C Annealed	1 MHz	−10	10	5.9256	6.0734
	500 kHz	−10	10	5.992	6.0545
	500 Hz	−10	10	112.50	490.74

**Table 2:** Optimal values of objective function using a TLBO technique for as-fabricated, 550 °C, and 950 °C annealed devices.

Device	Iteration	Value of Objective Function	*c* (F)	*V* (volt)	*E* (pj)
Charge storage for 500 Hz					
As fabricated	300	9.400000 × 10^−8^	1.88 × 10^−9^	10	94,000.00
550 °C	300	6.300670 × 10^−8^	1.26013 × 10^−9^	10	63,006.50
950 °C	300	2.453700 × 10^−8^	4.9074 × 10^−10^	−10	24,537.00
Charge storage for 500 KHz					
As fabricated	300	3.625900 × 10^−10^	7.2518 × 10^−12^	10	362.59
550 °C	300	3.449500 × 10^−10^	6.899 × 10^−12^	−10	344.95
950 °C	300	3.027250 × 10^−10^	6.0545 × 10^−12^	10	302.73
Charge storage for 1 MHz					
As fabricated	300	3.260335 × 10^−10^	6.52067 × 10^−12^	−10	326.03
550 °C	300	3.168324 × 10^−10^	6.33665 × 10^−12^	10	316.83
950 °C	300	3.036700 × 10^−10^	6.0734 × 10^−12^	10	303.67

## Data Availability

Not applicable.
